# Unbalanced Treatment Costs of Breast Cancer in China: Implications From the Direct Costs of Inpatient and Outpatient Care in Liaoning Province

**DOI:** 10.34172/ijhpm.2021.75

**Published:** 2021-08-01

**Authors:** Zihua Ma, Gongman Deng, Zhaolin Meng, Yanan Ma, Huazhang Wu

**Affiliations:** ^1^Department of Health Service Management, China Medical University, Shenyang, China.; ^2^The First Affiliated Hospital, College of Medicine, Zhejiang University, Hangzhou, China.; ^3^School of Nursing, Capital Medical University, Beijing, China.; ^4^Department of Biostatistics and Epidemiology, School of Public Health, China Medical University, Shenyang, China.

**Keywords:** Breast Cancer, Healthcare Expenditure, Medical Service Utilization, Cost Containment, China

## Abstract

**Background:** The increasing incidence of breast cancer and its financial burden highlights the need for controlling treatment costs. This study aimed to assess the direct costs of inpatient and outpatient care for breast cancer patients in Liaoning Province to provide a policy reference for cost containment.

**Methods:** Based on the System of Health Accounts 2011 (SHA 2011), systematic data collection was conducted via multistage stratified cluster random sampling. A total of 1160 health institutions, including 83 hospitals, 16 public health institutions, 120 primary health institutions, and 941 outpatient institutions were enrolled in 2017. A database was established containing 20 035 patient-level medical records from the information system of these institutions. Curative care expenditure (CCE)was calculated, and generalized linear modeling was performed to determine cost-related factors.

**Results:** In 2017, the CCE for breast cancer was approximately CNY 830.19 million (US$122.96 million) in Liaoning province (0.7% of the total health expenditure and 9.9% of cancer-related healthcare costs). Inpatient care costs were estimated to be CNY 617.27 million (US$91.42 million), accounting for 74.4% of the CCE for breast cancer, almost three times as large as outpatient costs (25.6%). The average inpatient and outpatient costs for breast cancer were estimated to be CNY 12 108 (US$1793) and CNY 829 (US$123) per visit. Medication cost was the main cost driver, which comprised 84.0% of the average outpatient cost and 37.2% of the mean inpatient cost.

**Conclusion:** Breast cancer imposes a large economic burden on patients and the social health insurance system. Results show an irrational cost pattern of inpatient and outpatient services, with patients relying excessively on inpatient services for treatment. Promoting outpatient care whenever relevant is conducive to cost containment and rational utilization of resources.

## Background

**Key Messages**

** Implications for policy makers**

- This study provides a comprehensive description of breast cancer healthcare cost based on the System of Health Accounts 2011 (SHA 2011), which will better understand the disease burden on the healthcare system, facilitate a more meaningful discussion of cost containment and allocation of resources.
- In 2017, the curative care expenditure (CCE) for breast cancer was about 830.19 million CNY in Liaoning province (0.7% of the total health expenditure and 9.9% of cancer-related healthcare costs), with out-of-pocket (OOP) payments accounting for 36.4% of total breast cancer care cost.
- Inpatient cost was the main cost driver (74.4%), almost three times as large as outpatient costs (25.6%).

** Implications for the public**

Providing affordable healthcare services so that anyone can get the required services is in accordance with the core idea of universal health coverage and the agenda of the Sustainable Development Goals. We are looking for options. This study indicates that there is an irrational cost pattern of inpatient and outpatient services in China, with patients relying excessively on inpatient services for treatment. This may increase the financial burden on patients and prolong the length of hospital stay. Thus, recommendations from international experience are provided to promote the transfer of cancer care from inpatient to outpatient settings whenever appropriate, which is conducive to cost containment and rational utilization of resources.

Breast cancer is the most commonly diagnosed cancer and the leading cause of cancer-related death among women worldwide. Globally, there were about 2.1 million cases of newly diagnosed female breast cancer in 2018, accounting for almost a quarter of cancer cases among women.^1^ In China, breast cancer is the most frequently diagnosed cancer (19.2%) and the fourth leading cause of cancer-related deaths among women (9.1%).^2^ The incidence of breast cancer among Chinese women has increased more than twice as fast as global rates. In 2018, with 367 900 new cases of invasive breast cancer and 97 972 related deaths, China accounted for 17.6% of global cases and 15.6% of related deaths, which is much higher than the proportion in 2008 (12.2% and 9.6%, respectively).^2,3^ Due to the high prevalence of breast cancer and the rapid increase in incidence, the costs related to the treatment of breast cancer will comprise a larger proportion of healthcare costs. Trends toward greater intensity of healthcare service use also lead to a greater economic burden of cancer in the future.

 The process of transforming cancer care from inpatient to outpatient settings has progressed far in many developed countries and produces significant savings in treatment.^4^ However, due to the policies to expand insurance coverage in China, the utilization of patient medical services has been greatly promoted, especially for inpatient services. The inpatient utilization rate has increased rapidly from 8.7% in 2008 to 17.6% in 2017.^5^ As a result, China has a higher inpatient utilization rate compared to the global rate (0.14 vs. 0.10 admissions per capita), while the outpatient utilization rate is still lower than the global rate (5.17 vs. 5.42 visits per capita).^6^ Since different patient service utilization patterns have a significant impact on the treatment cost of breast cancer, an analysis of inpatient and outpatient treatment costs associated with breast cancer will facilitate a more meaningful discussion of cost containment and allocation of resources.

 Several studies have assessed the direct medical costs of breast cancer in China,^7-9^ but to our knowledge none have studied the cost patterns of inpatient and outpatient services for breast cancer patients. Moreover, these studies are limited in that they focus merely on costs for inpatients or patients in specialized hospitals and lack the support of a systematic accounting framework and representative samples, thus decreasing the accuracy of expenditure calculation results and comparability with different countries.

 Measuring expenditure on breast cancer in a comparative and standard manner is a critical first step in understanding the economic burden of breast cancer. The System of Health Accounts 2011 (SHA 2011) is the most widely used approach for estimating health expenditures.^10-13^ It was revised and launched by several international organizations including the Organization for Economic Cooperation and Development, Eurostat, and the World Health Organization (WHO) and commonly used as the global standard in guiding systematic data collection or routine tracking health expenditures, which makes the calculated result comparable across countries.^14^ Furthermore, analyses based on the SHA 2011 can provide more comprehensive insights on tracking health expenditure through three interfaces that help explain the disease burdens on the healthcare system: the provider interface, the consumer health interface, and the financing interface.^15^

 This study aimed to estimate the curative care expenditure (CCE) for breast cancer in Liaoning Province, Northeast China, using the SHA 2011 framework, and assess the average inpatient and outpatient costs per visit based on the sample data. Combining the results of macro and micro data, we further evaluated the distribution of CCE and the cost patterns of inpatient and outpatient services to provide a policy reference for cost containment in China.

## Methods

###  Data Sources

**Figure 1:**
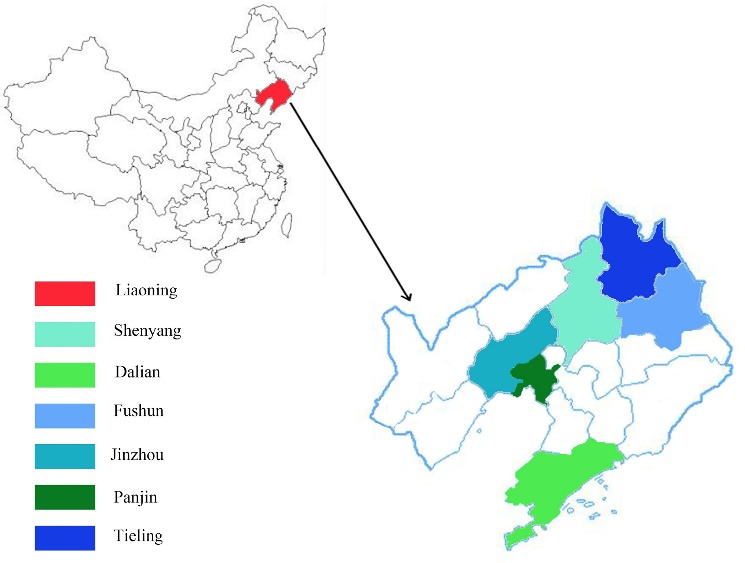
Locations of the Province and Cities Where the Study Was Done.

 For this population-based study, we selected patients in Liaoning Province as the sample population, and the macro data and demographic data were derived from official statistics and field investigations, respectively. There were about 44 million inhabitants and the gross domestic product was 234 090 billion in Liaoning Province. The total health expenditure was extracted from the Liaoning Health Statistical Yearbook (2017) and Liaoning Health Financial Yearbook (2017). The case data were gathered from sample institutions, which were selected via a multistage stratified cluster random sampling approach. In the first stage, sample cities were selected from Liaoning Province based on the economic development level and population density, and Shenyang, Dalian, Fushun, Jinzhou, Panjin, and Tieling were identified and selected as appropriate cities. Figure 1 indicated the locations of the province and cities where the study was done. In the second stage, one district and two counties were selected from each included prefecture-level city based on the quality of available data and the completeness of their health information systems. After determining the sample areas, in the third stage, a random sample was selected, ensuring that a representative sample of health agencies and hospital levels were included. A total of 1160 health institutions, including 83 hospitals, 16 public health institutions, 120 primary health institutions, and 941 outpatient institutions were enrolled. A database was established containing patient-level medical records from the information system of these institutions, including age, sex, date of hospital visit, primary diagnosis, length of stay (LOS), insurance type, and inpatient and outpatient costs.

###  Study Sample

 The study population comprised all patients with primary diagnosis as breast cancer of the facilities included in the study within the Liaoning province between January 1 and December 31, 2017. The International Classification of Disease Tenth Revision (ICD-10) was used to identify primary breast cancer, coded as C50. A total of 23 014 breast cancer-related medical records were selected from the database. After excluding records with missing values, suspected errors, and outliers (with the total cost below the 1st percentile or above the 99th percentile of the total cost distribution for inpatient and outpatient care), the final valid sample size for the overall breast cancer records was 20 035.

###  Measurement

####  Estimating CCE for Breast Cancer 

 Based on the SHA 2011, a top-down allocation method was used to calculate the CCE for breast cancer in Liaoning province. After extracting the total amount of current health expenditures from the macro data, the sharing coefficient calculated with the data from the field investigation was used to allocate the total health expenditures into various dimensions (such as age and institution type) and calculate the provincial breast cancer CCE. Age was defined in increments of 5-year periods, and institution type was defined as general hospitals, specialized hospitals, or traditional Chinese medicine (TCM) hospitals.

 In China, there are two major compensation sources for public hospitals, which are government subsidies and service charges. The government’s subsidies for public hospitals mainly includes two types: recurrent investment and special item investment. The recurrent investment is to subsidize the medical service, mainly determined according to the number of hospital staff and the number of beds provided, while the special item investment is to subsidize infrastructure, scientific research and the purchase of large-scale equipment. Therefore, the calculation of CCE mainly includes the regular investment of the government subsidies-that is, basic expenditure subsidies (BES), and the service charges of hospitals—that is, curative income (CI). The specific calculation formula is as follows:


$$S_{CCE}=S_{OCI}+S_{OBES}+S_{ICI}+S_{IBES}$$

In the above formula, S_OCI_ and S_OBES_ represent the CI and BES for outpatient service, and S_ICI_ and S_IBES_ represent the CI and BES for inpatient service.

 Since the calculation method of inpatient CCE is similar to that of outpatient CCE, we take outpatient CCE as an example to introduce the specific measures of accounting. To calculate the outpatient CI of the breast cancer, first, the total outpatient medical institution income was extracted through the summary of relevant data in the statistical yearbook and financial annual reports. Second, the proportion of prevention expenses was calculated from the sample data. Finally, the CI was calculated after deducting the total prevention expenses from the total hospital income. The formula for CI was as follows:


$$S_{OCI}=S_{TOMI}\left(1-\frac{\alpha_{p}}{\alpha }\right)$$

And CI for a certain dimension was calculated according to the following formula:


$$S_{OCI}{}^{\prime }=\sum_{i=1}^{n}\left(S_{OCI}\times \frac{a_{i}}{\alpha -\alpha_{p}}\right)$$

In the above two formulas, CI per visit from the sample data is denoted as *a*_i_, and total income of the sample hospitals is denoted as *a*. Moreover, the sample’s total preventive costs, denoted as *a*_p_, was calculated by adding all samples that contained preventive services based on the ICD-10 codes. Thus, the sharing coefficient for each patient is denoted by $a_{i}/\left(a-a_{p}\right)$ .

 The outpatient basic expenditure subsidy (OBES) was also calculated in 3 steps. First, the total BES, the total number of inpatient bed days (*b*), and the total number of outpatient visits (c) were extracted from the statistical yearbook and financial annual reports. Second, to separate preventive outpatient visits from the total outpatient visits (c), the total number of curative outpatient visits (d) was calculated based on the number of preventative outpatient visits (e) and total outpatient visits (f) in the sample data. Third, the hospitalization sharing coefficient (β) was calculated based on the total number of inpatient bed days (b) and the total number of curative outpatient visits (d) from the sample data. Finally, the OBES was calculated after deducting the inpatient BESs from the total BESs. The formulas for the BES are as follows:


$$S_{OBES}\;=\;S_{TOBES}\;\times \;\left(1\;–\;\beta \right)$$

$$\beta =\frac{b}{b+d*K}$$

Considering the conversion relationship between the workload of a physician for 1 hospital day and 1 outpatient visit, the value of K is set to 0.1 according to the recommendation of China National Health Development Research Center.


$$d=c\times \left(1-\frac{e}{f}\right)$$

The outpatient BESs for a certain dimension were calculated according to the following formula:


$$S_{OBES^{\prime }}=\sum_{i=1}^{n}\left(S_{OBES}\times \frac{\alpha_{i}}{\alpha -\alpha_{p}}\right)$$

We also calculated the CCE by three types of financing schemes: government financing schemes (eg, government subsidies, basic social health insurance), voluntary financing schemes (eg, non-government social insurance contributions, commercial health insurance payments, and donations from non-governmental organizations), and out-of-pocket (OOP) payments.^17^

####  Estimating Average Cost Per Inpatient and Outpatient Visit for Breast Cancer 

 The charges of each inpatient admission and outpatient visit associated with breast cancer were calculated based on the sample data from the field investigation. Descriptive statistics were used to determine demographic variables, healthcare resource utilization, and costs, including frequencies, percentage, means and standard deviation (SD), median, and 95% confidence intervals. Age at diagnosis was divided into 4 groups (<40, 40-54, 55-69, and ≥70 years) based on the age range of the target population for cancer screening in China (between 40 and 69 years of age).^18^ Moreover, patients’ insurance status was divided into four categories: self-paid and three main social health insurance schemes, including the Urban Employee Basic Medical Insurance (UEBMI), the Urban Resident Basic Medical Insurance (URBMI), and the New Cooperative Medical Scheme (NCMs). The inpatient costs consisted of the following components: prescription medication, imaging, laboratory examinations, therapy, surgery, materials, beds, diagnoses, and nursing care costs. Outpatient costs included prescription medication, imaging, laboratory examinations, therapy, surgery, materials, registration, and consultation fees.

 Following the descriptive analysis, the Mann-Whitney rank-sum test was used to determine the significance of observed differences in costs, and the chi-square statistic was used to compare proportions. To determine the cost-related factors of inpatient and outpatient costs, generalized linear modeling with a gamma distribution and a log link was performed. The generalized linear modeling model accounts for skewness in the distribution of medical costs without requiring retransformation of the results from the log scale back to levels.^19^ The cost-related factors in this study included age, institution type, insurance type, LOS, and surgery. Cost estimates that were different from zero at the 95% confidence level were considered to be statistically significant. All expense data were reported in Chinese yuan (CNY). The exchange rate between US dollar and CNY was: US$1.00 = CNY 6.7518 in 2017. The analyses were conducted using STATA 14.0 and SPSS 22.0 statistical software.

## Results

###  Characteristics of Inpatient and Outpatient Visits

 A total of 7416 inpatient admissions associated with breast cancer were included in the analyses. The mean ± SD age of the patients was 54.2 ± 10.4 years, and most of the admitted patients were 40-69 years old (85.8%). The average LOS was 9.8 ± 8.3 days. A total of 2704 patients (36.5%) underwent surgery. Most patients (92.7%) were supported by social health insurance. Patients were mainly admitted to general hospitals (67.6%), followed by TCM hospitals (16.9%) and specialist hospitals (15.4%).

 A total of 12 619 outpatient visits associated with breast cancer were assessed. The mean ± SD age of the patients was 58.1 ± 11.0 years. Similar to the age distribution of inpatients, 81.9% of outpatients were 40-69 years old. However, in contrast to the institution distribution of inpatients, 62.7% of cases were diagnosed or treated in specialized hospitals, and 32.2% in general hospitals. Moreover, the proportion of self-paying outpatients was high (20.1%), which is much higher than the proportion of self-paying inpatients (7.3%). Table 1 shows the characteristics of inpatient admissions and outpatient visits.

**Table 1:** Characteristics of Breast Cancer Inpatient Admission and Outpatient Visit

**Variable**	**Inpatient Admission**	**Outpatient Visit**
	**(n = 7416)**	**(n = 12619)**
Age, years (Mean, SD)	54.2 (10.4)	58.1 (11.0)
Age group (n, %)		
<40	582 (7.8)	657 (5.2)
40-54	3322 (44.8)	3770 (29.9)
55-69	3040 (41.0)	6565 (52.0)
≥70	472 (6.4)	1627 (12.9)
LOS (Mean, SD)	9.8 (8.3)	NA
Surgery	2704 (36.5)	88 (0.7)
Payment type (n, %)		
UEBMI	2561 (34.5)	2641 (20.9)
URBMI	2726 (36.8)	7170 (56.8)
NCMs	1589 (21.4)	277 (2.2)
Self-paid	540 (7.3)	2531 (20.1)
Hospital type (n, %)		
TCM hospital	1257 (16.9)	648 (5.1)
Specialized hospital	1144 (15.4)	7913 (62.7)
General hospital	5015 (67.6)	4058 (32.2)

Abbreviations: SD, standard deviation; UEBMI, Urban Employee Basic Medical Insurance; URBMI, Urban Resident Basic Medical Insurance; NCMs, New Rural Cooperative Medical Scheme; LOS, length of stay; TCM, Traditional Chinese Medicine.

###  Curative Care Expenditure for Breast Cancer 

 In 2017, the CCE for breast cancer was about CNY 830.19 million **(**US$122.96 million) in Liaoning province, which accounted for 0.7% of the total health-care expenditure and 9.9% of cancer-related healthcare costs. Inpatient care costs were estimated to be CNY 617.27 million **(**US$91.42 million), accounting for 74.4% of the CCE for breast cancer. Outpatient care costs were calculated to be CNY 212.92 million **(**US$31.54 million), accounting for 25.6% of the CCE for breast cancer. Of the three types of financing schemes, the government financing scheme provided the most, with CNY 429.28 million (51.7%). Of this, most funds came from basic social health insurance (41.9%), and government subsidies only accounted for 9.8%. The OOP payment for breast cancer patients was CNY 302.12 million **(**US$44.75 million), which accounted for 36.4% of the total CCE among breast cancer patients. Regarding different types of institutions, over 60% of the expenditure on treatment for breast cancer was spent in general hospitals, followed by specialist hospitals (22.8%) and TCM hospitals (10.6%).

**Figure 2:**
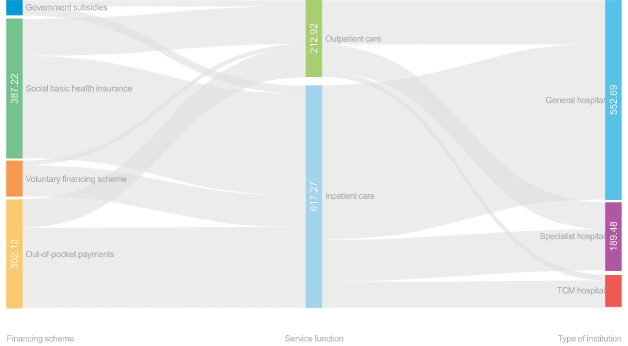
CCE Associated With Breast Cancer by Financing Scheme, Service Function, and Type of Institution. Abbreviation: CCE, Curative Care Expenditure.

 Similar to the total CCE pattern, the money paid by social health insurance and OOP payments went more to inpatient services (73.0%), whereas the proportion paid by government subsidies was directed slightly less to inpatient care (57.0%). Among different types of hospitals, the share of inpatient and outpatient care costs was slightly different. TCM hospitals and general hospitals spent significantly more on inpatient services (82.2% vs. 77.9%), while inpatient and outpatient costs in specialized hospitals were more balanced (59.9% vs. 40.1%). Figure 2 shows how the CCE for breast cancer was simultaneously split by financing schemes, service use (inpatient admissions and outpatient visits), and type of institution.

**Figure 3:**
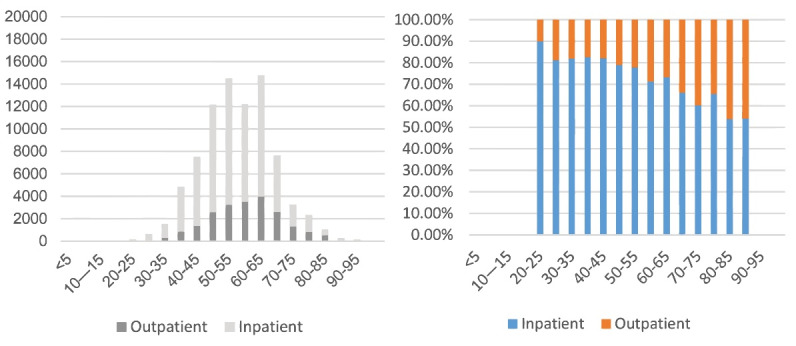
The Age Distribution of Breast CCE for Inpatient Admissions and Outpatient Visits. Abbreviation: CCE, Curative Care Expenditure.

Figure 3 displays the age distribution of breast cancer CCE for inpatient admissions and outpatient visits. The CCE for breast cancer was dominated by the population aged 40-69 years, accounting for 82.9%, with a small portion of costs among patients under 40 years or over 70 years. The total CCE for breast cancer, including outpatient and inpatient CCEs, all showed a trend of rising first and then falling with age. The age distribution had two peaks, at 50-54 years old and 60-64 years old, accounting for 17.5% and 17.8% of the total CCE, respectively. The breast cancer CCE for inpatient services was mostly from people aged 50–54 years, accounting for 18.3% of the total inpatient CCE. The 60-64 year old age group accounted for the highest proportion (18.6%) of the total outpatient CCE. In addition, inpatient CCE always accounted for the majority of the total costs in all age groups (over 50.0%), but as age increased, its proportion gradually decreased.

###  Charges From Each Inpatient Admission and Outpatient Visit

 Resource utilization for both inpatient care and outpatient care per visit is presented in Table 2. The average inpatient and outpatient costs for breast cancer were estimated to be CNY 12 108 (US$1793) and CNY 829 (US$123) per visit, respectively. The average inpatient cost per admission was about 14.6 times the average outpatient cost per visit. The main cost drivers for inpatient cost were medication expense and material consumption, which accounted for 37.2% and 19.1%, respectively, while the proportions for surgical treatment (10.0%) and therapy expenses (6.8%) were relatively low. The major source of expenses for outpatient services was medication, which contributed to the bulk of the total outpatient costs (84.0%), while other cost categories only comprised small portions of the total costs.

**Table 2:** Cost Category of Mean Inpatient and Outpatient Cost Per Visit (CNY)

**Cost Category**	**Inpatient**		**Outpatient**	
	**Mean ± SD**	**Percentage of Cost (%)**	**Mean ± SD**	**Percentage of Cost (%)**
Total	12108 ± 10629	100.0	829 ± 505	100.0
Medication	4502 ± 4326	37.2	696 ± 545	84.0
Imaging	1605 ± 2356	13.3	58 ± 198	7.0
Laboratory test	1029 ± 899	8.5	25 ± 105	3.0
Therapy	826 ± 1893	6.8	12 ± 114	1.4
Surgery	1209 ± 2036	10.0	3 ± 37	0.4
Material	2312 ± 4647	19.1	20 ± 73	2.4
Bed	335 ± 630	2.8	NA	NA
Diagnose	111 ± 133	0.9	NA	NA
Nursing	180 ± 228	1.5	NA	NA
Registration	NA	NA	10 ± 43	1.2
Consultation	NA	NA	5 ± 49	0.6

Abbreviations: CNY, Chinese Yuan; SD, standard deviation. The exchange rate between US dollar and CNY was: US$1.00 = CNY 6.7518 in 2017.

###  Predictors of Inpatient and Outpatient Costs

 The factors related to inpatient and outpatient costs per visit for breast cancer, are presented in Supplementary file 1. For inpatient services, univariate analysis shows that age, surgery, insurance type, and hospital type are highly correlated the cost per admission (all *P*< .001, Table S1). The multivariable analysis adjusted for these factors indicates that young age, undergoing surgery, having insurance, and admission in a TCM hospital were independently associated with high costs (Table S2). Moreover, both univariate and multivariate analyses showed that patients supported by insurance with better benefits such as UEBMI (with higher funding criteria and reimbursement rates) had higher costs.

 Slightly different from inpatient costs, although the factors related to the outpatient costs per visit were age, insurance status, and hospital type (all *P*< .001), surgery was non-significant in the univariate analysis (Table S1). Furthermore, multivariate analysis showed that older age, visit to a specialist or TCM hospital, and having insurance (excluding NCMs) were independently associated with high costs (Table S2).

## Discussion

 Our study provides much-needed data on the scale and distribution of CCEs associated with breast cancer. We found that the direct expenditure of breast cancer is burdensome and cost patterns of inpatient and outpatient services are irrational compared to those in developed countries.

 The CCE for breast cancer in Liaoning Province in 2017 accounted for 9.9% of cancer-related healthcare costs, which is a higher percentage than the national average level. According to Global Cancer Statistics 2018, breast cancer incidence cases account for 367 900 cases out of 4 285 033 cancer-related cases (C00-C97) in China, corresponding a share of 8.6%. Moreover, the high prevalence of breast cancer and its rapid increase in incidence have brought a heavy economic burden to the health insurance system and individual patients. Our results show that the funds for breast cancer treatment mainly came from basic social health insurance (46.0%) and OOP payments (36.4%), with little voluntary financing and government subsidies. Despite China’s commitment and progress in reducing the medical financial burden on households and ensuring access to healthcare, our results show that the financial burden on the individual for breast cancer is still much higher than the government’s preset goal, which is to reduce the percentage of the OOP payments in total health expenditure to below 28% by 2020.^17^ Moreover, the mean inpatient cost per visit for breast cancer was 12 108 CNY, accounting for 46.6% of the average annual disposable income per capital in Liaoning in 2017, further suggesting that the financial burden on the individual for breast cancer care is excessive. Relying too much on OOP payments may result in catastrophic health expenditure and impoverishment from medical expenses. Therefore, relevant policies are urgently needed to control the growth of breast cancer costs and reduce the personal medical burden of patients.

 Our results show that of the total CCE for breast cancer, inpatient cost was the main cost driver (74.4%). These results are different from those reported in other countries, which show that the major cost component of breast cancer care is outpatient cost, accounting for 71% of the total costs in the United States and 62% in France.^21-23^ The relatively high proportion of inpatient CCE may indicate irrational utilization patterns of inpatient and outpatient services for breast cancer care, and patients relying too much on inpatient services. There are two possible reasons for the over-utilization of inpatient services. First, this may be related to the reimbursement system of medical insurance. According to Zhang et al, China’s medical insurance schemes are mainly intended to provide financial protection for inpatients, while the coverage of outpatient services is very limited or not covered at all.^24^ Our results show that the proportion of self-paid outpatients was significantly higher than that of self-paid inpatients (20.1% vs. 7.3%). As patients mainly rely on inpatient services for reimbursement, patients are more inclined to choose inpatient services to receive treatment, thus further exacerbating the imbalance between the proportion of outpatients and inpatients. Second, this may be related to the relatively smaller number of service items provided in outpatient settings. Our results showed that medication expenses comprised about 84.0% of the outpatient costs, which suggests that the service function of outpatient care is relatively simple, mainly for prescription drugs, while more complex treatment services are generally obtained through hospitalization.

 Moreover, the average hospital stay for breast cancer in China (9.8 days) is much longer compared to the United States and many European Union countries, with an average LOS of 5.7 days and 4.2-5.0 days, respectively.^25,26^ The magnitude of inpatient costs and longer hospital stays highlight the importance of considering shifting the medical services from inpatient to outpatient settings. Chemotherapy is one of the main treatments for breast cancer. In China, approximately 81.4% of breast cancer patients receive chemotherapy, and most of them are treated in an inpatient setting,^3^ which is different from in many developed countries. Recently, outpatient chemotherapy has become a relatively popular international practice. Patient-centered daily chemotherapy wards have been widely implemented in many countries to improve the efficiency of medical resources and reduce costs. A previous study showed that the transition of chemotherapy to an outpatient setting led to a greater than 20% reduction in inpatient oncology costs.^27^ The benefits of this shift also include reducing the need for inpatient medical resources, decreasing hospital stays, reducing infection rates, and improving quality of life.^25^ According to Joo et al, patients who begin chemotherapy administration in an outpatient setting report greater satisfaction with their treatment compared with those treated in an inpatient setting.^28^ Therefore, outpatient chemotherapy and surgery should be promoted to facilitate more non-essential hospitalization patients transferring to outpatient settings, thereby reducing the economic burden of patients.

 We found that breast cancer costs were concentrated among people aged 40–69, which is mainly related to the age of onset of breast cancer. Although inpatient costs consistently contributed to the majority of the total costs in all age groups, younger patients had a higher proportion of resource consumption for inpatient services than the older groups. Furthermore, per capita, the cost was significantly higher for younger inpatients and older outpatients. These results may indicate that younger patients are more dependent on inpatient services for aggressive treatment than older patients. However, excessive hospitalization services could impose a higher financial burden and lead to more loss of working time. In China, the median age of breast cancer diagnosis is much younger than in western countries, with over half of cases diagnosed before 50 years of age.^3,29^ This means that the majority of women who are diagnosed are still of working age, which may suggest that the loss of productivity in China is greater than that in western countries. A comparative study between Chinese and American cancer survivors confirmed this point. The study showed that Chinese cancer survivors are more likely than American cancer survivors to retire earlier than planned (37% vs. 9%) and reported more hindrance to work abilities due to cancer treatment (84% vs. 21%).^30^ With outpatient treatment, patients can return to work more easily during treatment with fewer side effects.^4^ Therefore, the transition from inpatient to outpatient treatment should be strengthened, especially for younger patients.

 Our results show that the CCE for breast cancer is mainly spent in general hospitals (66.6%), other than specialist hospitals (22.8%), which may be associated with the insufficient and uneven distribution of specialist medical resources. Although more targeted in cancer treatment, specialized hospitals have limited beds and are mostly concentrated in provincial capitals. Additionally, our results show that inpatient and outpatient costs in specialized hospitals are more balanced (59.9% and 40.1%) compared to general hospitals and TCM hospitals. One of the possible reasons is that the inpatient reception capacity is limited by the number of beds in a specialist hospital, so many non-essential inpatients are transferred to outpatient treatment. Conversely, outpatient reception capacity is not restricted by the number of beds, so specialist hospitals can receive more outpatients, which is verified by the fact that a higher percentage of outpatients visit specialist hospitals (62.7%), and a higher percentage of inpatients visit general hospitals (67.6%) (Table 1). The effective use of outpatient services for specialist hospitals is conducive to controlling the total medical expenses in the region. Therefore, as the main provider of breast cancer care, the transfer of inpatient services to outpatient settings should be focused on general hospitals to control the total cost.

 We found that several factors influence the health expenditure of patients based on the univariate and multivariate analyses, including age, surgery, LOS, type of hospital, and insurance status. The impact of some factors on costs is difficult to control through policies, while the impact of certain factors, such as the LOS and insurance status, can be regulated. As in other studies,^31^ we found that costs were significantly higher for patients supported by health insurance than self-paying ones. In addition, patients under an insurance scheme with higher funding criteria and reimbursement rates (such as UEBMI) had higher costs. The reason may be related to the fact that medical insurance promotes healthcare utilization. Based on the fee-for-service payment, both doctors and patients are motivated to use more medical services, thus resulting in higher healthcare costs. Therefore, China has launched a series of reforms by moving from fee-for-service to diagnosis-related groups, which was initially proven to be effective in reducing hospital expenditures and LOS.^32^ Furthermore, the diagnosis-related group policy can effectively control hospitalization expenses and promote the transfer of medical services to outpatient settings. Therefore, the wider application of diagnosis-related groups should be strengthened.

 This study had several limitations. First, since the individual outpatient and inpatient records were reported by the sample hospital with the patient identification anonymized, we could not determine the number of cancer patients, the number of outpatient visits and inpatients admissions per patient in 2017, and the annual per capital cost of patients with breast cancer. Second, our database had no further diagnostic information, and we could not determine the cancer stage of the patients. Although cancer staging has a significant impact on the cost of breast cancer care, we were unable to conduct a stratified analysis of patients with different cancer stages. Third, due to the lack of comprehensive data on indirect costs, our analysis is limited to direct costs. Due to the above reasons, our estimates may underestimate the overall medical service cost of cancer treatment. Despite these limitations, healthcare costs were obtained from the SHA, which includes a representative sample used to provide a comprehensive and comparative cost analysis for breast cancer care.

## Conclusion

 Breast cancer imposes a significant economic burden on both patients and the social health insurance system in Liaoning Province. In addition, there are irrational cost patterns of inpatient and outpatient service use, with an overreliance on inpatient services. Specific policies are needed to promote the transfer of cancer care from inpatient to outpatient settings, which is conducive to cost containment and rational utilization of resources. Lessons learned, recommendations from international experience, and evidence from the systematic data can inform decisions about the allocation of resources to service provision and support policy-makers in reducing the financial burden on society and patients in China and other countries with similar situations.

## Ethical issues

 The need for ethics approval was waived by the Ethics Committee of China Medical University, Shenyang, China, on the basis that the data used in this study is anonymized and de-identified. No identified or potentially identifiable human information was collected or generated in this study.

## Competing interests

 Authors declare that they have no competing interests.

## Authors’ contributions

 The contributions made by the individual authors are as follows. ZHM, YNM, and HZW designed the study; HZW coordinated the data collection; ZHM and GMD conducted data analysis and interpretation; ZHM wrote the first draft; ZLM, YNM, and HZW provided data analysis recommendations and revised the ﬁnal manuscript. All authors read and approved the manuscript.

## Disclaimer

 The authors declare that the research was conducted in the absence of any commercial or financial relationships that could be construed as a potential conflict of interest.

## Funding

 This paper was supported by the Education Department of Liaoning Province under Grant No: LZDR201701.

## Supplementary files

Supplementary file 1 contains Tables S1 and S2.
