# Supplementary material for: Unbalanced Treatment Costs of Breast Cancer in China: Implications From the Direct Costs of Inpatient and Outpatient Care in Liaoning Province
Source: Int J Health Policy Manag. 2021 Aug 1;11(9):1735–43. doi: 10.34172/ijhpm.2021.75 (PMC9808230; doi:10.34172/ijhpm.2021.75)
Supplement: Supplementary file 1 — contains Tables S1 and S2. [file ijhpm-11-1735-s001.pdf]

Article title: Unbalanced Treatment Costs of Breast Cancer in China: Implications from the Direct Costs of Inpatient and Outpatient Care in Liaoning Province

Journal name: International Journal of Health Policy and Management (IJHPM)

Authors' information: Zihua Ma[1], Gongman Deng[2], Zhaolin Meng[3], Yanan Ma[4]*, Huazhang Wu[1]*

[1]Department of Health Service Management, China Medical University, Shenyang, China.

[2]The First Affiliated Hospital, College of Medicine, Zhejiang University, Hangzhou, China.

[3]School of Nursing, Capital Medical University, Beijing, China.

[4]Department of Biostatistics and Epidemiology, School of Public Health, China Medical University, Shenyang, China.

(*corresponding author: ynma@cmu.edu.cn & hzwu@cmu.edu.cn)

Supplementary file 1.

Table S1. Average expenditure (CNY) per visit for inpatient and outpatient care

	Inpatient cost			Outpatient cost		
	Mean	Median	P value	Mean	Median	P value
Age group			<0.001			<0.001
<40	13305	10799		641	362	
40-54	12973	9810		698	468	
55-69	11122	8863		890	1201	
≥70	10899	8134		960	1213	
Surgery			<0.001			0.442
NO	7775	5817		829	826	
YES	19659	17614		807	767	
Insurance status			<0.001			<0.001
UEBMI	13265	9903		948	1213	
URBMI	11853	8353		856	739	
NCMs	11350	8267		687	495	
Self-paid	10162	7220		643	401	
Hospital type			<0.001			<0.001
TCM hospital	12306	9704		814	674	
Specialized hospital	15263	15159		914	1213	
General hospital	11339	7575		665	412	
TOTAL	12108	8699		829	824	

Abbreviation: UEBMI, Urban Employee Basic Medical Insurance; URBMI, Urban Resident Basic Medical Insurance; NCMs, New Rural Cooperative Medical Scheme; TCM hospital, Traditional Chinese Medicine hospital.

Table S2. Regression results for generalized linear model of inpatient and outpatient costs per visits

	Analysis 1: Inpatient cost			Analysis 2: Outpatient cost		
	coefficient	95% CI		coefficient	95% CI	
Age group						
<40	0.1	0.033	0.167	-0.343	-0.405	-0.28
40-54	0.047	0.006	0.101	-0.264	-0.304	-0.223
55-69	0.02	-0.033	0.074	-0.079	-0.115	-0.042
≥70	Reference			Reference		
LOS	0.05	0.049	0.052	NA	NA	NA
Surgery	0.76	0.79	0.73	0.126	-0.019	0.271
Payment type						
UEBMI	0.362	0.308	0.416	0.231	0.198	0.263
URBMI	0.306	0.252	0.36	0.168	0.083	0.252
NCMs	0.24	0.185	0.295	0.014	-0.057	0.029
Self-paid	Reference			Reference		
Hospital type						
TCM hospital	0.382	0.339	0.426	0.151	0.09	0.211
Specialized hospital	0.03	-0.009	0.068	0.159	0.123	0.194
General hospital	Reference			Reference		

Abbreviation: UEBMI, Urban Employee Basic Medical Insurance; URBMI, Urban Resident Basic Medical Insurance; NCMs, New Rural Cooperative Medical Scheme; TCM hospital, Traditional Chinese Medicine hospital.
